# Disruption of Slc4a10 augments neuronal excitability and modulates synaptic short-term plasticity

**DOI:** 10.3389/fncel.2015.00223

**Published:** 2015-06-16

**Authors:** Anne Sinning, Lutz Liebmann, Christian A. Hübner

**Affiliations:** ^1^Institute of Human Genetics, University Hospital Jena, Friedrich Schiller University JenaJena, Germany; ^2^Institute of Physiology, University Medical Center of the Johannes Gutenberg UniversityMainz, Germany

**Keywords:** Slc4a10, field potential, GABAergic inhibition, synaptic plasticity, LTP

## Abstract

Slc4a10 is a Na^+^-coupled Cl^−^-HCO_3_^−^ exchanger, which is expressed in principal and inhibitory neurons as well as in choroid plexus epithelial cells of the brain. Slc4a10 knockout (KO) mice have collapsed brain ventricles and display an increased seizure threshold, while heterozygous deletions in man have been associated with idiopathic epilepsy and other neurological symptoms. To further characterize the role of Slc4a10 for network excitability, we compared input-output relations as well as short and long term changes of evoked field potentials in Slc4a10 KO and wildtype (WT) mice. While responses of CA1 pyramidal neurons to stimulation of Schaffer collaterals were increased in Slc4a10 KO mice, evoked field potentials did not differ between genotypes in the stratum radiatum or the neocortical areas analyzed. Paired pulse facilitation was diminished in the hippocampus upon disruption of Slc4a10. In the neocortex paired pulse depression was increased. Though short term plasticity is modulated via Slc4a10, long term potentiation appears independent of Slc4a10. Our data support that Slc4a10 dampens neuronal excitability and thus sheds light on the pathophysiology of SLC4A10 associated pathologies.

## Introduction

Proper brain function depends on a well-balanced interplay between excitation and inhibition. Disturbing this balance can cause severe neurological disorders like epilepsy. GABA is the main inhibitory neurotransmitter in the central nervous system, which mainly acts via GABA_A_ and GABA_B_ receptors. While the latter are metabotropic receptors that are linked to potassium channels via G-proteins, GABA_A_ receptors are ligand-gated anion channels that mainly conduct chloride and bicarbonate at physiological conditions. In the developing brain the Na^+^-K^+^-Cl^−^ co-transporter NKCC1 and AE3/Slc4a3 (Hentschke et al., 2006; Pfeffer et al., 2009) accumulate chloride into neurons. Thus, opening of GABA_A_ receptors causes a depolarizing efflux of chloride, which is deemed to be important for the development of the neuronal circuitry (Ben-Ari, 2002). With the incipient expression of the neuronal K^+^/Cl^−^ co-transporter KCC2, i.e., in the second postnatal week in rodents the chloride gradient reverses (Rivera et al., 1999; Stein et al., 2004). As a consequence the opening of GABA_A_ receptors typically results in a hyperpolarizing influx of chloride, which is the correlate of fast synaptic inhibition.

The bicarbonate gradient always results in a depolarizing efflux of bicarbonate upon opening of GABA_A_ receptors (Rivera et al., 2005; Farrant and Kaila, 2007; Hübner and Holthoff, 2013). In neurons with a rather hyperpolarized resting membrane potential and a low intracellular chloride concentration this current can exceed the chloride current and thus result in bicarbonate dependent depolarization (Kaila and Voipio, 1987; Hübner and Holthoff, 2013). However, the role of bicarbonate and hence the role of neuronal mechanisms to control intracellular bicarbonate levels are often neglected. In neurons, bicarbonate transport is mainly mediated by members of the SLC4A family of proteins. While the Na^+^-independent anion-exchanger SLC4A3 lowers the intraneuronal bicarbonate concentration, the Na^+^-dependent anion exchangers SLC4A8 (NDCBE) and SLC4A10 (NCBE) use the sodium gradient to accumulate bicarbonate in exchange of chloride (Chesler, 2003; Romero et al., 2013). The raise in the intracellular bicarbonate concentration may augment the depolarizing efflux of bicarbonate upon activation of GABA_A_ receptors; however, both transporters also extrude chloride and thereby increase the gradient for a hyperpolarizing chloride current. Moreover, the transport of bicarbonate is inseparably linked to changes in pH with consequences for both neuronal excitability and synaptic transmission (Chesler and Kaila, 1992; Sinning and Hübner, 2013). Thus, it is quite difficult to predict the consequences of the disruption of either SLC4A8 or SLC4A10 on neuronal excitability.

Although in mice both transporters are broadly expressed within the brain (Hübner et al., 2004; Damkier et al., 2007; Chen et al., 2008; Sinning et al., 2011), there are some notable differences: while Slc4a8 is restricted to excitatory principal neurons, Slc4a10 also localizes to inhibitory neurons (Jacobs et al., 2008). Slc4a8 is enriched in presynaptic nerve terminals (Sinning et al., 2011; Burette et al., 2012) and supports glutamate release in a pH-dependent manner. As a consequence Slc4a8-deficient mice display an increased seizure threshold (Sinning et al., 2011). Slc4a10-knockout mice also have an increased seizure threshold, however, the neurological phenotype is more complex and includes visual impairment (Hilgen et al., 2012) and collapsed brain ventricles (Jacobs et al., 2008). The latter is most likely owed to a compromised production of the cerebrospinal fluid, because Slc4a10 is prominently expressed in choroid plexus epithelial cells (Praetorius et al., 2004). Surprisingly, different neurological disorders including idiopathic epilepsy have been associated with heterozygous deletions of large genomic regions spanning the human SLC4A10 (Gurnett et al., 2008; Krepischi et al., 2010; Belengeanu et al., 2014).

Aim of the present study was to better characterize the role of Slc4a10 for neuronal excitability and plasticity of synaptic connections in different brain regions. Extracellular field recordings of acute brain slice preparations revealed an increase of somatic field responses and a lower paired pulse ratio in the hippocampal CA1 region of Slc4a10 KO mice, while long-term potentiation (LTP) in response to tetanic stimulation was not changed. In the visual and auditory cortex, synaptic short term plasticity was modulated, but amplitudes of evoked field responses were unchanged. No genotype-dependent differences in LTP induced by tetanic stimulation were noted in the cortex. Taken together, these data suggest that Slc4a10 plays an important, so far unknown role as a modulator of synaptic short term plasticity in different neocortical areas. Slc4a10 dampens the excitability of CA1 pyramidal neurons and may thus act as a regulator of the excitation/inhibition balance in the brain.

## Materials and Methods

### Animals

All experiments were approved by the responsible local institutions and complied with the regulations of the National Institutes of Health and those of the Society of Neuroscience (Washington, DC, USA). Constitutive knockout (KO) mice were generated as described earlier (Jacobs et al., 2008). In brief, deletion of exon 12 of the *Slc4a10* gene, which encodes for the first of the predicted transmembrane spans of Slc4a10, leads to a frame shift and a premature stop codon in exon 13. Total KO and *Slc4a10* wild type (WT) mice were generated from heterozygous matings in a pure C57/Bl6 background. Mice were group-housed on a 12 h light–dark cycle and fed with food and water *ad libitum*. For all experiments we used littermates with a 50/50 gender ratio from heterozygous matings.

### Slice Preparation for Electrophysiological Recordings

400-μm-thick coronal brain slices were prepared from 2–3-month-old mice and equilibrated in aCSF (in mM: 120 NaCl, 3.5 KCl, 1.3 MgSO_4_, 1.25 NaH_2_PO_4_, 2.5 CaCl_2_, 10 D-glucose, 25 NaHCO_3_; gassed with 95% O_2_/5% CO_2_, pH 7.3) at RT for at least 1 h, as described previously (Liebmann et al., 2008). Slices were transferred to an interface recording chamber and perfused with oxygenated aCSF (2–3 ml/min) at 34°C. For LTP recordings, horizontal brain slices and a slightly modified aCSF (in mM: 124 NaCl, 3 KCl, 1.5 MgSO_4_, 1.25 NaH_2_PO_4_, 2 CaCl_2_, 10 D-glucose, 24 NaHCO_3_) were used to prevent the occurrence of spreading depressions or epileptiform discharges after high frequency stimulation.

### Cortical and Hippocampal Field Potential Recordings: Paired Pulse Paradigm

Evoked field potentials were investigated on coronal brain sections by placing of bipolar stimulating electrodes with a tip separation of 100 μm (SNE-200X, Science-Products, Germany) onto layer VI of the cortex or the Schaffer collaterals of the hippocampal CA3 area, respectively. Upon stimulation (pulse duration 50 μs), field potentials were recorded using glass microelectrodes (impedance 2–5 MΩ, filled with aCSF) impaled into the cortical layer II/III of the cortex. In the hippocampus evoked field potentials were recorded simultaneously from the stratum radiatum and the stratum pyramidale of the CA1 area as described previously (Sinning et al., 2011). For all experiments stimulus intensity was gradually increased until responses saturated (0–70 V) and the half-maximal stimulus intensity was determined (inter-stimulus interval 30 s). After determination of the half-maximal stimulus intensity, paired-pulse stimuli were applied with inter-stimulus intervals of 15, 20, 30, 50, 80, 120, 180, 280, 430, 650 and 1000 ms.

Data of field potential recordings were collected with an extracellular amplifier (EXT-02, NPI, and Germany), low pass filtered at 4 kHz and digitally stored with a sample frequency of 10 kHz. Analysis of field potential recordings was performed using the software “Signal” (Cambridge Electronic Design, UK). Absolute amplitudes and slopes were analyzed for population spikes (PS) and population synaptic responses (fEPSP), respectively. To assess fEPSP-PS coupling, slopes of fEPSP recorded in the stratum radiatum and the amplitudes of the simultaneously recorded respective PS in the stratum pyramidale were correlated. For the comparison between genotypes mean PS amplitudes within fEPSP slope bins of 0.5 mV/ms were calculated.

### Cortical and Hippocampal Field Potential Recordings: LTP

For hippocampal LTP recordings, Schaffer collaterals were stimulated in the CA3 region of the hippocampus by a bipolar stimulation electrode (PI2ST30.1A3, tip separation 75 μm, Science Products, Germany). Recordings were performed from the stratum radiatum and the stratum pyramidale of the hippocampal CA1 region and fEPSP amplitudes and PS slopes analyzed, respectively. For cortical LTP, stimulation electrodes were placed in layer VI of the cortex and recordings were performed in cortical layer II/III. After determination of the half maximal stimulus intensity a stable baseline of responses was recorded for 20 min with a stimulation frequency of 0.05 Hz. LTP was induced by repeated high frequency stimulation with half-maximal stimulus intensity (2 × 100 pulses at 100 Hz, inter stimulus interval 1 s) in the hippocampus and in the cortex (5 × 100 pulses at 100 Hz). Evoked responses were recorded subsequently for 60 min with a stimulus frequency of 0.05 Hz. Slopes of hippocampal fEPSPs and PS and cortical fEPSP amplitudes were normalized to its mean during baseline recording.

### Immunohistochemistry

Free-floating cryosections (50 μm) were stained with a polyclonal rabbit anti-Slc4a10 antibody (Jacobs et al., 2008). For co-stainings, the following primary antibodies were used: polyclonal guinea pig anti-vesicular GABA transporter (VGAT, 1:500, Synaptic Systems) and polyclonal guinea pig anti-GABA_A_ receptor subunit α5 (1:4000; Redecker et al., 2000). Alexa Fluor 488- and 555-coupled goat anti-rabbit and goat anti-guinea pig antibodies were used as secondary antibodies (1:1000, Invitrogen). Analysis was performed using an inverted fluorescence microscope equipped with an ApoTome (Cell Observer, Zeiss).

### Statistical Analysis

Data are presented as mean ± SEM. Statistical analysis of two experimental groups was performed with the parametric two tailed Student’s *t*-test. In experiments that included repeated measurements, differences between groups were tested by repeated-measures ANOVA. If applicable, subsequent Bonferroni *post hoc* tests were applied. Significance was considered at *p* values <0.05.

## Results

### Disruption of Slc4a10 Increases Somatic Field Potentials in the Hippocampus

**Figure 1:**
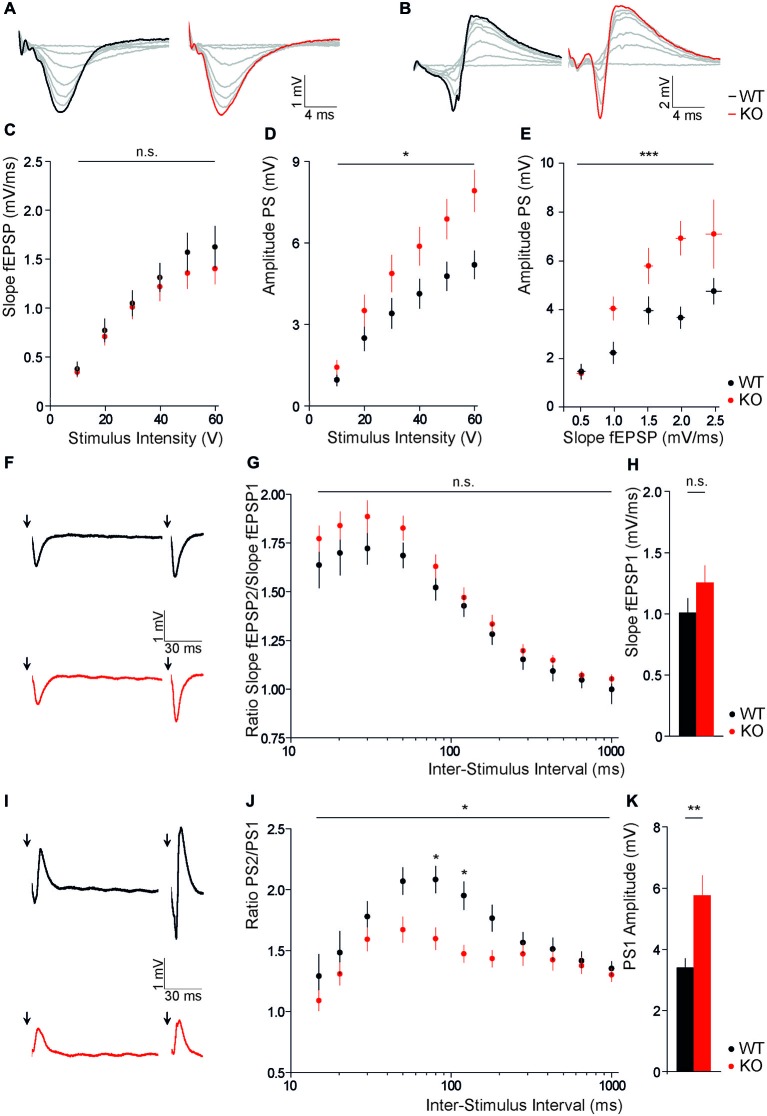
**Disruption of Slc4a10 increases somatic field potentials in the hippocampus. (A,B)** Representative examples of fEPSPs recorded in the stratum radiatum **(A)** and stratum pyramidale **(B)** in WT and KO slices upon stimulation of Schaffer collaterals with increasing stimulus intensity (0–60 V), respectively. **(C)** Slopes of fEPSPs recorded in the stratum radiatum of CA1 are independent of Slc4a10 (*p* > 0.05; *n* = 20/19). **(D)** CA1 population spike amplitudes upon stimulation of Schaffer collaterals were increased in acute brain slices of Slc4a10 KO mice (*p* = 0.04; *n* = 22/27). **(E)** The correlation between the slope of fEPSP recorded in the stratum radiatum and the amplitude of PS in the stratum pyramidale revealed a significant increase in spike coupling (*p* < 0.001, *n* = 14/14). **(F)** Exemplary traces of fEPSP recorded in the stratum radiatum **(F)** upon repeated stimulation of Schaffer collaterals with an inter-stimulus interval of 120 ms in WT and KO slices. **(G)** Disruption of Slc4a10 had no impact on dendritic paired-pulse facilitation (*p* > 0.05; *n* = 18/17). **(H)** Mean fEPSP slopes upon stimulation with half-maximal stimulus intensity did not differ between genotypes (*p* > 0.05; *n* = 18/17). **(I)** Exemplary responses of fEPSP recorded in the stratum pyramidale upon repeated stimulation of Schaffer collaterals with an inter-stimulus interval of 120 ms in WT and KO slices. **(J)** Paired-pulse facilitation at an inter-stimulus interval of 80 and 120 ms was decreased (*p* = 0.04; *n* = 18/22). **(K)** Note, at half-maximal stimulus intensity the mean first PS amplitude also showed a significant increase in KO slices. (*p* = 0.001; *n* = 18/22). *p < 0.05 **p < 0.005 ***p < 0.001.

To assess whether the disruption of Slc4a10 affects network excitability, we recorded evoked field potentials in different regions of acute brain slices of Slc4a10 KO and WT mice and analyzed input-output relationships. Firstly, evoked extracellular field responses of CA1 pyramidal neurons to a single stimulation of the Schaffer collaterals were analyzed. Whereas slopes of fEPSP recorded in the stratum radiatum did not differ between genotypes (Figures 1A,C; repeated-measures ANOVA, *F* = 0.47; *p* = 0.50), PS amplitudes recorded in the stratum pyramidale were increased in Slc4a10 KO mice (Figures 1B,D; repeated-measures ANOVA, *F* = 4.16; *p* = 0.04). Half-maximal stimulation intensities did not differ between genotypes (KO 39.4 ± 6.8 V; WT 32.8 ± 5.1 V; *n* = 27/22; Student’s *t*-test *p* = 0.50). Next, we correlated slopes of evoked fEPSP with the respective population spike amplitudes. This analysis revealed that a larger population spike amplitude in KO mice manifests with a more efficient coupling between the synaptically driven fEPSP-slope and the action-potential derived PS-amplitude in the hippocampus (Figure 1E; repeated-measures ANOVA, *F* = 8.52; *p* < 0.0001). These results suggest an increased excitability of CA1 pyramidal neurons and a positive shift in fEPSP/PS coupling efficiency in Slc4a10 KO mice.

### Slc4a10 Modulates Synaptic Short Term Plasticity in the Hippocampus

For the analysis of short term plasticity, we applied paired stimuli with a varying inter-stimulus interval (15–1000 ms) to Schaffer collaterals and compared paired pulse ratios between genotypes. In recordings from the stratum radiatum of slices from Slc4a10 KO mouse brains we did not observe significant changes in the paired pulse ratios of the slopes of fEPSP2 and fEPSP1 (Figures 1F,G; repeated-measures ANOVA, *F* = 1.38; *p* = 0.25). In agreement with the unaltered input-output relationship, there was also no change in the slope of the response to the first half-maximal stimulus (Figure 1H; KO: 1.25 ± 0.15 mV; WT: 1.00 ± 0.12 mV; *n* = 18/17; Student’s *t*-test *p* = 0.21). In the stratum pyramidale, however, the PS amplitude at half-maximal stimulus intensity was increased (Figure 1K; KO: 5.75 ± 0.65 mV; WT: 3.39 ± 0.30 mV; *n* = 18/22; Student’s *t*-test *p* = 0.001), while the paired pulse ratio was decreased (Figures 1I,J; repeated-measures ANOVA, *F* = 5.12; *p* = 0.03) upon disruption of Slc4a10. The Bonferroni *post hoc* analysis revealed that the genotype-dependent difference in paired pulse ratio only applies to an inter-stimulus interval of 80 and 120 ms (80 ms: KO 1.60 ± 0.09, WT 2.08 ± 0.11; *n* = 18/22; Bonferroni *post hoc* test *p* < 0.05; 120 ms: KO 1.47 ± 0.07, WT 1.95 ± 0.11; *n* = 18/22; Bonferroni *post hoc* test *p* < 0.05). Thus, paired Schaffer collateral stimulations showed that the increase in somatic excitability of CA1 hippocampal neurons is accompanied by a decreased paired pulse ratio in the stratum pyramidale.

### Slc4a10 Deletion does not Affect Long-Term Potentiation in the Hippocampus

**Figure 2:**
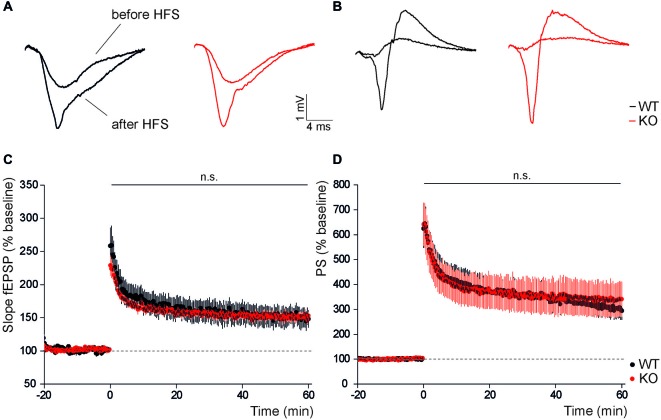
**In the hippocampus long-term potentiation is not affected by disruption of Slc4a10. (A,B)** Representative examples of dendritic and somatic field responses recorded in the hippocampal stratum radiatum and stratum pyramidale of WT and KO slices before and after high frequency stimulation of Schaffer collaterals.** (C)** Dendritic LTP recorded in the stratum radiatum of CA1 after high frequency stimulation is unaltered in KO slices (*p* > 0.05; *n* = 11/11). **(D)** Similarly, no genotype-dependent difference was observed in the potentiation of the population spike amplitude recorded in the stratum pyramidale after high frequency stimulation (*p* > 0.05; *n* = 11/11).

We next assessed whether disruption of Slc4a10 also affects hippocampal long-term plasticity. For this purpose we stimulated Schaffer collaterals with a tetanic stimulation and analyzed evoked postsynaptic potentials of CA1 pyramidal neurons. We compared early (averaged changes to baseline from 0 min to 5 min) and late (averaged changes to baseline from 55 min to 60 min) potentiation of postsynaptic excitability after two trains of 100 pulses at 100 Hz. Normalized field responses in the stratum radiatum were likewise increased after high frequency stimulation but there was no difference between genotypes (Figures 2A,C; early potentiation: KO 193.0 ± 7.2%, WT 178.6 ± 19.0%, *n* = 11/11, Student’s *t*-test *p* = 0.81; late potentiation: KO 149.0 ± 5.8%, WT 140.6 ± 12.7%, *n* = 11/11, Student’s *t*-test *p* = 0.53). Also, LTP recorded from the stratum pyramidale did not differ between genotypes, neither in the early phase (Figures 2B,D; KO 517.4 ± 78.2%, WT 529.7 ± 68.7%, *n* = 11/11, Student’s *t*-test *p* = 0.91), nor in the late phase (KO 338.4 ± 69.4%, WT 304.4 ± 38.3%, *n* = 11/11, Student’s *t*-test *p* = 0.67). Taken together, these results do not support an important role of Slc4a10 for the induction or maintenance of LTP in the hippocampus.

### Slc4a10 is Expressed in Cortical GABAergic Synapses and Modulates Synaptic Short Term Plasticity in the Cortex

**Figure 3:**
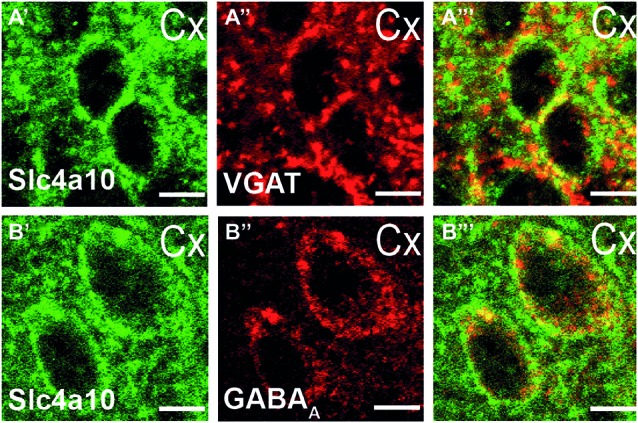
**Synaptic expression of Slc4a10 in the cortex. (A′–A′′′)** Overlap of Slc4a10 and VGAT, a marker for GABAergic presynapses (green: Slc4a10, red: VGAT). **(B′–B′′′)** Slc4a10 co-localizes also with the postsynaptic GABA_A_-receptor in cortical neurons (green: Slc4a10, red: GABA_A_- α5).

Slc4a10 is abundantly expressed in the somatodendritic compartment of hippocampal as well as cortical principal neurons (Jacobs et al., 2008; Song et al., 2014). To specify whether Slc4a10 localizes to synapses we performed double immunostainings of WT brain slices for Slc4a10 and different synaptic markers. In the visual cortex we found a substantial co-localization of Slc4a10 with VGAT, a marker of GABAergic presynapses (Figure 3A′–A′′′), and the GABA_A_ receptor subunit α5, as a marker for GABAergic postsynapses (Figure 3B′–B′′′).

**Figure 4:**
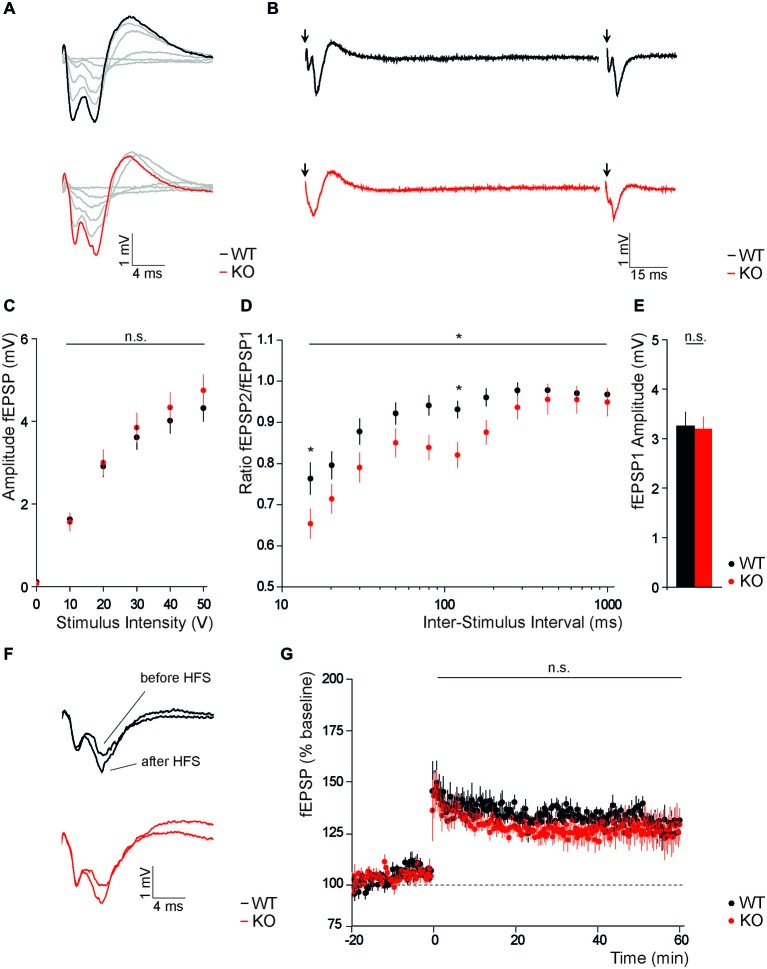
**Disruption of Slc4a10 affects short term plasticity in the occipital and temporal cortex. (A,B)** Representative traces of fEPSPs recorded in occipital and temporal cortical areas from WT and KO slices upon single columnar stimulations with increasing stimulus intensity **(A)** and paired stimulation with an interstimulus interval of 120 ms **(B)**. **(C)** Absolute fEPSP amplitudes recorded in occipital and temporal cortical areas did not differ between genotypes (*p* > 0.05; *n* = 44/52). **(D)** The paired-pulse ratio was significantly decreased in brains slices of KO mice. This was most obvious at an inter-stimulus interval of 15 and 120 ms (*p* = 0.015; *n* = 40/50). **(E)** No difference was observed for the mean first fEPSP amplitude upon half-maximal stimulation (*p* > 0.05; *n* = 40/50). **(F)** Exemplary traces of fEPSPs recorded in cortical layers 2/3 of WT and KO slices before and after columnar high frequency stimulation. **(G)** LTP recordings from WT and KO slices did not reveal genotype dependent differences in the potentiation of cortical fEPSP amplitudes after high frequency stimulation. *p < 0.05.

To assess the role of Slc4a10 for excitability and plasticity in cortical neurons, we recorded evoked field responses of layer 2/3 neurons in response to stimulation of cortical layer 6 in both the visual and the auditory cortex. No differences were observed in field potential amplitudes, neither in response to stimulation with an increasing stimulus intensity (Figures 4A,C; *n* = 44/52 repeated-measures ANOVA, *F* = 0.62; *p* = 0.24), nor in the average response to repetitive stimulation with half-maximal stimulus intensity (Figure 4E; KO 3.26 ± 0.26, WT 3.39 ± 027; *n* = 40/50; Student’s *t*-test *p* = 0.85). Paired pulse stimulation revealed a significantly decreased paired pulse ratio in KO slices (Figures 4B,D; *n* = 40/50; repeated-measures ANOVA, *F* = 6.21; *p* = 0.015). *Post hoc* analysis revealed that genotype-dependent differences only applied to inter-stimulus intervals of 15 and 120 ms (15 ms: KO 0.65 ± 0.03, WT 0.76 ± 0.03; 120 ms: KO 0.82 ± 0.03, WT 0.93 ± 0.03, KO; *n* = 40/50; Bonferroni *post hoc* test *p* < 0.05).

These results suggest that Slc4a10 co-localizes with pre- and postsynaptic markers of GABAergic synapses in the cortex and modulates synaptic short-term but not long-term plasticity in the cortex.

### Slc410 does not Affect Long-Term Potentiation in the Cortex

Finally, we addressed the functional role of Slc4a10 on long term plasticity in the cortex and compared absolute amplitudes of synaptic population responses evoked in cortical layers 2/3 upon tetanic stimulation within cortical layer 6 of WT and KO slices. Normalized field responses were increased upon high frequency stimulation in both groups (Figure 4F), but comparison of potentiation in the early and late phase of LTP did not reveal differences between genotypes (Figures 4F,G; early potentiation: KO 135.4 ± 5.5%, WT 139.5 ± 7.9% *n* = 6/5, Student’s *t*-test *p* = 0.84; late potentiation: KO 123.1 ± 7.9%, WT 124.5 ± 4.9%, *n* = 6/5, Student’s *t*-test *p* = 0.89).

Thus, these results suggest that also in the cortex Slc4a10 does not have an important functional role neither for the induction nor for the maintenance of LTP.

## Discussion

Here, we show that disruption of Slc4a10 increases the excitability of CA1 pyramidal neurons in the somatic but not the dendritic compartment. Paired pulse facilitation of PS was decreased in the stratum pyramidale, while it was not changed in the stratum radiatum. Short-term plasticity was also altered in different cortical areas, while amplitudes of evoked field potentials did not differ between genotypes. Hippocampal and cortical LTP were not changed in Slc4a10 KO mice.

### Sub-Regional Differences in Slc4a10-Dependent Alterations of Single Evoked Field Potentials in the Hippocampus

Field potentials recorded in different compartments of the hippocampal CA1 region have different electrophysiological origins: while there is a graded dendritic potential in the stratum radiatum, the action-potential based population spike recorded from the stratum pyramidale originates at the axon hillock. Increased amplitudes of field potentials in the stratum pyramidale in Slc4a10 KO mice indicate that the expression of Slc4a10 decreases the likelihood of action potential generation for a defined excitatory postsynaptic potential, a phenomenon that is commonly described as excitatory postsynaptic potential-or EPSP-spike coupling. Such a potentiation of CA1 response which is also supported by a positive shift in fEPSP/PS-spike coupling efficiency in KO slices can either be caused by a change in the intrinsic excitability of pyramidal neurons or by impaired GABAergic inhibition (Daoudal et al., 2002; Staff and Spruston, 2003). While there is considerable GABAergic input to the stratum pyramidale, it is sparse in the distal stratum radiatum. Consequently, changes in GABAergic inhibition are more likely to affect somatic excitability (Megías et al., 2001). Because of the strong expression of Slc4a10 in hippocampal interneurons (Jacobs et al., 2008; Song et al., 2014), changed PS, unaltered fEPSP slopes in the stratum radiatum and a positive shift in the fEPSP/PS-spike coupling efficiency may be indicative for a compromised GABAergic inhibition upon disruption of Slc4a10.

### Slc4a10 Affects Short-Term but not Long-Term Plasticity at Hippocampal Synapses

Multiple forms of short-term plasticity including facilitation and depression co-occur at synapses (Raimondo et al., 2012). Nevertheless, short-term plasticity of hippocampal synaptic connections is dominated by facilitation. Facilitation is the consequence of residual presynaptic calcium after the conditioning pulse, which transiently increases transmitter release probability (Katz and Miledi, 1968; Zucker and Regehr, 2002). At short intervals (<50 ms) the response to the second stimulus is limited via GABA_A_ dependent feed forward inhibition, while at intervals between 100–125 ms activation of presynaptic GABA_B_ autoreceptor activation dominates (Davies et al., 1990; Steffensen and Henriksen, 1991). The decrease in paired-pulse facilitation at 80 and 120 ms interstimulus intervals is thus compatible with a modulation of GABA_B_ receptor mediated inhibition in the hippocampus in Slc4a10 KO mice.

In contrast to the hippocampus, short-term plasticity at neocortical synapses is dominated by depression (Deisz and Prince, 1989; Markram and Tsodyks, 1996), which is of multimodal origin (Zucker and Regehr, 2002). Besides classical depletion of vesicle pools, a reduced paired pulse ratio in the cortex is mainly attributed to activation of GABA_B_ receptors (Takesian et al., 2010). The reduction of paired pulse ratios in the cortex of Slc4a10 KO mice at either 15 or 120 ms is consistent with a modulation of both GABA_A_ and GABA_B_ receptor mediated inhibition in Slc4a10 KO mice (Davies et al., 1990; Wehr and Zador, 2005).

Based on the alterations in short-term plasticity upon disruption for Slc4a10, we expected enhanced LTP in Slc4a10 KO slices. However, Slc4a10 deletion did not alter the levels of LTP in the hippocampus, neither when recorded in the stratum radiatum nor in the stratum pyramidale, and also not in the cortex. Long-lasting changes in postsynaptic excitability such as LTP in principal CA1 hippocampal neurons require AMPA-receptor mediated activation of postsynaptic NMDA-receptors (Bliss and Collingridge, 1993) and structural changes at synapses (Engert and Bonhoeffer, 1999; Malenka and Bear, 2004). GABAergic inhibition has a profound effect on the depolarization of the postsynaptic neuron and hence NMDA-receptor activation in response to the tetanic stimulation (Wigström and Gustafsson, 1983; Lu et al., 2000). Nevertheless, the influence of GABAergic inhibition on LTP of excitatory responses appears to be stimulation-dependent (Chapman et al., 1998) and can occur in the absence of both inhibitory and excitatory GABAergic signaling (Debray et al., 1997). Under conditions of high-frequency stimulation GABA_B_ autoreceptor mediated suppression of GABA release is known to promote the induction of LTP (Davies et al., 1991). However, these effects may be blunted in Slc4a10 KO mice.

### Outlook

While K^+^/Cl^−^ co-transporters have been extensively studied for their role in regulating neuronal excitability under physiological and pathophysiological conditions (Blaesse et al., 2009), the role of Slc4 bicarbonate transporters in regulation of neuronal excitability and synaptic activity has mostly been neglected. In light of the evidence that bicarbonate transporters of the SLC4A family including SLC4A10 are involved in seizure disorders (Gurnett et al., 2008; Krepischi et al., 2010; Belengeanu et al., 2014), a better understanding of their role for synaptic transmission is desirable to get a more comprehensive view of neuronal excitability and the pathophysiology of epilepsy (Chesler, 2003; Leniger et al., 2004).

## Conflict of Interest Statement

The authors declare that the research was conducted in the absence of any commercial or financial relationships that could be construed as a potential conflict of interest.

## References

[B1] Belengeanu V. Gamage T. H. Farcas S. Stoian M. Andreescu N. Belengeanu A.. (2014). A de novo 2.3 Mb deletion in 2q24.2q24.3 in a 20-month-old developmentally delayed girl. Gene 539, 168–172. 10.1016/j.gene.2014.01.060 24508274

[B2] Ben-Ari Y. (2002). Excitatory actions of GABA during development: the nature of the nurture. Nat. Rev. Neurosci. 3, 728–739. 10.1038/nrn920 12209121

[B3] Blaesse P. Airaksinen M. S. Rivera C. Kaila K. (2009). Cation-chloride cotransporters and neuronal function. Neuron 61, 820–838. 10.1016/j.neuron.2009.03.003 19323993

[B4] Bliss T. V. Collingridge G. L. (1993). A synaptic model of memory: long-term potentiation in the hippocampus. Nature 361, 31–39. 10.1038/361031a0 8421494

[B5] Burette A. C. Weinberg R. J. Sassani P. Abuladze N. Kao L. Kurtz I. (2012). The sodium-driven chloride/bicarbonate exchanger in presynaptic terminals. J. Comp. Neurol. 520, 1481–1492. 10.1002/cne.22806 22102085 PMC3856893

[B6] Chapman C. A. Perez Y. Lacaille J. C. (1998). Effects of GABA_A_ inhibition on the expression of long-term potentiation in CA1 pyramidal cells are dependent on tetanization parameters. Hippocampus 8, 289–298. 10.1002/(sici)1098-1063(1998)8:3<289::aid-hipo10>3.0.co;2-x 9662142

[B7] Chen L. M. Kelly M. L. Parker M. D. Bouyer P. Gill H. S. Felie J. M.. (2008). Expression and localization of Na-driven Cl-/HCO_3_^−^ exchanger (SLC4A8) in rodent CNS. Neuroscience 153, 162–174. 10.1016/j.neuroscience.2008.02.018 18359573 PMC2905791

[B8] Chesler M. (2003). Regulation and modulation of pH in the brain. Physiol. Rev. 83, 1183–1221. 10.1152/physrev.00010.2003 14506304

[B9] Chesler M. Kaila K. (1992). Modulation of pH by neuronal activity. Trends Neurosci. 15, 396–402. 10.1016/0166-2236(92)90191-a 1279865

[B10] Damkier H. H. Nielsen S. Praetorius J. (2007). Molecular expression of SLC4-derived Na^+^-dependent anion transporters in selected human tissues. Am. J. Physiol. Regul. Integr. Comp. Physiol. 293, R2136–R2146. 10.1152/ajpregu.00356.2007 17715183

[B11] Daoudal G. Hanada Y. Debanne D. (2002). Bidirectional plasticity of excitatory postsynaptic potential (EPSP)-spike coupling in CA1 hippocampal pyramidal neurons. Proc. Natl. Acad. Sci. U S A 99, 14512–14517. 10.1073/pnas.222546399 12391303 PMC137914

[B12] Davies C. H. Davies S. N. Collingridge G. L. (1990). Paired-pulse depression of monosynaptic GABA-mediated inhibitory postsynaptic responses in rat hippocampus. J. Physiol. 424, 513–531. 10.1113/jphysiol.1990.sp018080 2167975 PMC1189826

[B13] Davies C. H. Starkey S. J. Pozza M. F. Collingridge G. L. (1991). GABA autoreceptors regulate the induction of LTP. Nature 349, 609–611. 10.1038/349609a0 1847993

[B14] Debray C. Diabira D. Gaiarsa J. L. Ben-Ari Y. Gozlan H. (1997). Contributions of AMPA and GABA_A_ receptors to the induction of NMDAR-dependent LTP in CA1. Neurosci. Lett. 238, 119–122. 10.1016/s0304-3940(97)00865-3 9464634

[B15] Deisz R. A. Prince D. A. (1989). Frequency-dependent depression of inhibition in guinea-pig neocortex *in vitro* by GABA_B_ receptor feed-back on GABA release. J. Physiol. 412, 513–541. 10.1113/jphysiol.1989.sp017629 2557431 PMC1190589

[B16] Engert F. Bonhoeffer T. (1999). Dendritic spine changes associated with hippocampal long-term synaptic plasticity. Nature 399, 66–70. 10.1038/19978 10331391

[B17] Farrant M. Kaila K. (2007). The cellular, molecular and ionic basis of GABA_A_ receptor signalling. Prog. Brain Res. 160, 59–87. 10.1016/s0079-6123(06)60005-8 17499109

[B18] Gurnett C. A. Veile R. Zempel J. Blackburn L. Lovett M. Bowcock A. (2008). Disruption of sodium bicarbonate transporter SLC4A10 in a patient with complex partial epilepsy and mental retardation. Arch. Neurol. 65, 550–553. 10.1001/archneur.65.4.550 18413482

[B19] Hentschke M. Wiemann M. Hentschke S. Kurth I. Hermans-Borgmeyer I. Seidenbecher T.. (2006). Mice with a targeted disruption of the Cl^−^/HCO_3_^−^ exchanger AE3 display a reduced seizure threshold. Mol. Cell. Biol. 26, 182–191. 10.1128/mcb.26.1.182-191.2006 16354689 PMC1317631

[B20] Hilgen G. Huebner A. K. Tanimoto N. Sothilingam V. Seide C. Garrido M. G.. (2012). Lack of the sodium-driven chloride bicarbonate exchanger NCBE impairs visual function in the mouse retina. PLoS One 7:e46155. 10.1371/journal.pone.0046155 23056253 PMC3467262

[B21] Hübner C. A. Hentschke M. Jacobs S. Hermans-Borgmeyer I. (2004). Expression of the sodium-driven chloride bicarbonate exchanger NCBE during prenatal mouse development. Gene Expr. Patterns 5, 219–223. 10.1016/j.modgep.2004.08.002 15567717

[B22] Hübner C. A. Holthoff K. (2013). Anion transport and GABA signaling. Front. Cell. Neurosci. 7:177. 10.3389/fncel.2013.00177 24187533 PMC3807543

[B23] Jacobs S. Ruusuvuori E. Sipilä S. T. Haapanen A. Damkier H. H. Kurth I.. (2008). Mice with targeted Slc4a10 gene disruption have small brain ventricles and show reduced neuronal excitability. Proc. Natl. Acad. Sci. U S A 105, 311–316. 10.1073/pnas.0705487105 18165320 PMC2224208

[B24] Kaila K. Voipio J. (1987). Postsynaptic fall in intracellular pH induced by GABA-activated bicarbonate conductance. Nature 330, 163–165. 10.1038/330163a0 3670401

[B25] Katz B. Miledi R. (1968). The role of calcium in neuromuscular facilitation. J. Physiol. 195, 481–492. 10.1113/jphysiol.1968.sp008469 4296699 PMC1351674

[B26] Krepischi A. C. Knijnenburg J. Bertola D. R. Kim C. A. Pearson P. L. Bijlsma E.. (2010). Two distinct regions in 2q24.2–q24.3 associated with idiopathic epilepsy. Epilepsia 51, 2457–2460. 10.1111/j.1528-1167.2010.02742.x 21204806

[B27] Leniger T. Thöne J. Bonnet U. Hufnagel A. Bingmann D. Wiemann M. (2004). Levetiracetam inhibits Na^+^-dependent Cl^−^/HCO_3_^−^ exchange of adult hippocampal CA3 neurons from guinea-pigs. Br. J. Pharmacol. 142, 1073–1080. 10.1038/sj.bjp.0705836 15249428 PMC1575181

[B28] Liebmann L. Karst H. Sidiropoulou K. van Gemert N. Meijer O. C. Poirazi P.. (2008). Differential effects of corticosterone on the slow afterhyperpolarization in the basolateral amygdala and CA1 region: possible role of calcium channel subunits. J. Neurophysiol. 99, 958–968. 10.1152/jn.01137.2007 18077660

[B29] Lu Y. M. Mansuy I. M. Kandel E. R. Roder J. (2000). Calcineurin-mediated LTD of GABAergic inhibition underlies the increased excitability of CA1 neurons associated with LTP. Neuron 26, 197–205. 10.1016/s0896-6273(00)81150-2 10798404

[B30] Malenka R. C. Bear M. F. (2004). LTP and LTD: an embarrassment of riches. Neuron 44, 5–21. 10.1016/j.neuron.2004.09.012 15450156

[B31] Markram H. Tsodyks M. (1996). Redistribution of synaptic efficacy between neocortical pyramidal neurons. Nature 382, 807–810. 10.1038/382807a0 8752273

[B32] Megías M. Emri Z. Freund T. Gulyás A. (2001). Total number and distribution of inhibitory and excitatory synapses on hippocampal CA1 pyramidal cells. Neuroscience 102, 527–540. 10.1016/s0306-4522(00)00496-6 11226691

[B33] Pfeffer C. K. Stein V. Keating D. J. Maier H. Rinke I. Rudhard Y.. (2009). NKCC1-dependent GABAergic excitation drives synaptic network maturation during early hippocampal development. J. Neurosci. 29, 3419–3430. 10.1523/JNEUROSCI.1377-08.2009 19295148 PMC6665272

[B34] Praetorius J. Nejsum L. N. Nielsen S. (2004). A SCL4A10 gene product maps selectively to the basolateral plasma membrane of choroid plexus epithelial cells. Am. J. Physiol. Cell Physiol. 286, C601–C610. 10.1152/ajpcell.00240.2003 14592810

[B35] Raimondo J. V. Markram H. Akerman C. J. (2012). Short-term ionic plasticity at GABAergic synapses. Front. Synaptic Neurosci. 4:5. 10.3389/fnsyn.2012.00005 23087642 PMC3472547

[B36] Redecker C. Luhmann H. J. Hagemann G. Fritschy J. M. Witte O. W. (2000). Differential downregulation of GABA_A_ receptor subunits in widespread brain regions in the freeze-lesion model of focal cortical malformations. J. Neurosci. 20, 5045–5053. 10864962 10.1523/JNEUROSCI.20-13-05045.2000 PMC6772268

[B37] Rivera C. Voipio J. Kaila K. (2005). Two developmental switches in GABAergic signalling: the K^+^-Cl^−^ cotransporter KCC2 and carbonic anhydrase CAVII. J. Physiol. 562, 27–36. 10.1113/jphysiol.2004.077495 15528236 PMC1665491

[B38] Rivera C. Voipio J. Payne J. A. Ruusuvuori E. Lahtinen H. Lamsa K.. (1999). The K^+^/Cl^−^ co-transporter KCC2 renders GABA hyperpolarizing during neuronal maturation. Nature 397, 251–255. 9930699 10.1038/16697

[B39] Romero M. F. Chen A. P. Parker M. D. Boron W. F. (2013). The SLC4 family of bicarbonate HCO_3_^−^ transporters. Mol. Aspects Med. 34, 159–182. 10.1016/j.mam.2012.10.008 23506864 PMC3605756

[B40] Sinning A. Hübner C. A. (2013). Minireview: pH and synaptic transmission. FEBS Lett. 587, 1923–1928. 10.1016/j.febslet.2013.04.045 23669358

[B41] Sinning A. Liebmann L. Kougioumtzes A. Westermann M. Bruehl C. Hübner C. A. (2011). Synaptic glutamate release is modulated by the Na^+^-driven Cl^−^/HCO_3_^−^ exchanger Slc4a8. J. Neurosci. 31, 7300–7311. 10.1523/JNEUROSCI.0269-11.2011 21593314 PMC6622604

[B42] Song X. Yamasaki M. Miyazaki T. Konno K. Uchigashima M. Watanabe M. (2014). Neuron type- and input pathway-dependent expression of Slc4a10 in adult mouse brains. Eur. J. Neurosci. 40, 2797–2810. 10.1111/ejn.12636 24905082

[B43] Staff N. P. Spruston N. (2003). Intracellular correlate of EPSP-spike potentiation in CA1 pyramidal neurons is controlled by GABAergic modulation. Hippocampus 13, 801–805. 10.1002/hipo.10129 14620875

[B44] Steffensen S. C. Henriksen S. J. (1991). Effects of baclofen and bicuculline on inhibition in the fascia dentata and hippocampus regio superior. Brain Res. 538, 46–53. 10.1016/0006-8993(91)90374-5 1850318

[B45] Stein V. Hermans-Borgmeyer I. Jentsch T. J. Hübner C. A. (2004). Expression of the KCl cotransporter KCC2 parallels neuronal maturation and the emergence of low intracellular chloride. J. Comp. Neurol. 468, 57–64. 10.1002/cne.10983 14648690

[B46] Takesian A. E. Kotak V. C. Sanes D. H. (2010). Presynaptic GABA_B_ receptors regulate experience-dependent development of inhibitory short-term plasticity. J. Neurosci. 30, 2716–2727. 10.1523/JNEUROSCI.3903-09.2010 20164356 PMC3842473

[B47] Wehr M. Zador A. M. (2005). Synaptic mechanisms of forward suppression in rat auditory cortex. Neuron 47, 437–445. 10.1016/j.neuron.2005.06.009 16055066

[B48] Wigström H. Gustafsson B. (1983). Facilitated induction of hippocampal long-lasting potentiation during blockade of inhibition. Nature 301, 603–604. 10.1038/301603a0 6298626

[B49] Zucker R. Regehr W. (2002). Short-term synaptic plasticity. Annu. Rev. Physiol. 64, 355–405. 10.1146/annurev.physiol.64.092501.114547 11826273

